# Glycometabolic Alterations in Secondary Adrenal Insufficiency: Does Replacement Therapy Play a Role?

**DOI:** 10.3389/fendo.2018.00434

**Published:** 2018-08-03

**Authors:** Chiara Graziadio, Valeria Hasenmajer, Mary A. Venneri, Daniele Gianfrilli, Andrea M. Isidori, Emilia Sbardella

**Affiliations:** Department of Experimental Medicine, Sapienza University of Rome, Rome, Italy

**Keywords:** secondary adrenal insufficiency, insulin resistance, weight gain, body mass index, metabolic syndrome, impaired glucose tolerance, diabetes mellitus, glucocorticoids

## Abstract

Secondary adrenal insufficiency (SAI) is a potentially life-threatening endocrine disorder due to an impairment of corticotropin (ACTH) secretion from any process affecting the hypothalamus or pituitary gland. ACTH deficit can be isolated or associated with other pituitary failures (hypopituitarism). An increased mortality due to cardiovascular, metabolic, and infectious diseases has been described in both primary and secondary adrenal insufficiency. However, few studies have provided compelling evidences on the underlying mechanism in SAI, because of the heterogeneity of the condition. Recently, some studies suggested that inappropriate glucocorticoid (GCs) replacement therapy, as for dose and/or timing of administration, may play a role. Hypertension, insulin resistance, weight gain, visceral obesity, increased body mass index, metabolic syndrome, impaired glucose tolerance, diabetes mellitus, dyslipidemia have all been associated with GC excess. These conditions are particularly significant when SAI coexists with other pituitary alterations, such as growth hormone deficiency, hypogonadism, and residual tumor. Novel regimen schemes and GC preparations have been introduced to improve compliance and better mimick endogenous cortisol rhythm. The controlled trials on the improved replacement therapies, albeit in the short-term, show some beneficial effects on cardiovascular risk, glucose metabolism, and quality of life. This review examines the current evidence from the available clinical trials investigating the association between different glucocorticoid replacement therapies (type, dose, frequency, and timing of treatment) and glycometabolic alterations in SAI.

## Introduction

Secondary adrenal insufficiency (SAI) is a rare potentially life-threatening condition due to any disturbance involving the pituitary gland (space-occupying lesions, trauma or genetic abnormalities) and interfering with corticotropin (ACTH) secretion (1).

The impairment of ACTH secretion can be isolated or can be associated with other defected pituitary secretions (hypopituitarism) (1). Studies on outcomes in hypopituitary patients have shown that the frequency of SAI is between 41 and 94% (2). SAI shows a prevalence of 150–280 per million with a higher prevalence in women (3).

Moreover iatrogenic suppression of the hypothalamic–pituitary–adrenal axis caused by long-term glucocorticoid exposure should be carefully considered and patients taking long-term oral, inhaled, intranasal, intra-articular, or topical glucocorticoids, should be assessed for possible hypocortisolism (4).

Several studies have demonstrated an increased mortality in patients with cortisol deficiency attributable to cardiovascular, metabolic, neoplastic and infective diseases (5). The incidence of adrenal crises is increasing, at an estimated 3–6 % per year among SAI, with a dramatic 1% mortality rate (5).

On the basis of the Endocrine Society Clinical Practice guidelines, the management of patients with adrenal insufficiency (AI) consists in treatment with hydrocortisone (HC) or cortisone acetate (CA), which are the most physiological choice for glucocorticoid replacement. The recommended daily hydrocortisone dose is 10–12 mg/m^2^ divided in two to three doses, administering half to two thirds of the total daily dose in the morning, generally suggesting lower daily dosage in SAI (1, 6). However, optimal glucocorticoid replacement therapy for adrenally insufficient patients is still controversial due to lack of evidence from randomized trials.

One of the main concerns in the management of AI is balancing the need to increase the usual glucocorticoid (GC) dose during stress conditions. The goal is the prevention of adrenal crises on one side, with the need to reduce over-exposure in the uneventful daily life. Even when administered according to current guidelines, multiple daily doses of glucocorticoids (md-GC) have been associated with bone loss and reduced quality of life (7, 8) probably from a higher time exposure in the evening (9).

Hypercortisolism is associated to hyperglycaemia and insulin resistance (IR), increasing glicogenolisis, and hepatic gluconeogenesis and playing a direct role on pancreatic insulin secretion (10). Phosphatidylinositol-3 kinase pathway is involved in insulin signaling reducing nitric oxide synthase activation and causing endothelial impairment, ultimately increasing cardiovascular risk (11). Hypertension, IR, weight gain, visceral obesity, increased body mass index (BMI), metabolic syndrome, impaired glucose tolerance, diabetes mellitus, dyslipidemia are associated with GC excess or unphysiological GC replacement in patients with SAI (12). All these complications are more relevant when SAI coexists with other pituitary disorders, such as neoplasms of the sellar region, growth hormone deficiency (GHD), hypogonadism, and all the subsequent comorbidities that commonly affect the hypopituitary patient (2, 13).

A rare but interesting cause of SAI is the homozygous or composed heterozygous loss-of-function mutations of POMC gene, leading to early onset adrenal insufficiency, obesity, and altered pigmentation (14). Given the pivotal role of POMC in regulating appetite and metabolism, whether the lack of POMC secretion in all-causes SAI can play a role in metabolic complications has never been investigated and should prompt further studies.

Recently, modified-release preparations have been developed to mimic the physiological cortisol rhythm and improve patients' compliance (15, 16). The once-daily modified-release hydrocortisone tablet (od-MRHC) has been introduced to prevent the afternoon peaks given by conventional therapies administered in multiple daily doses. Treatment with the od-MRHC seems to improve cardiovascular risk factors, glucose metabolism, and quality of life in controlled trials (17–19).

The objective of this review is to evaluate the association between inadequate glucocorticoid replacement therapy (type, dose, frequency, and timing of treatment) and glycometabolic alterations in SAI, on the basis of currently available data.

## Glucocorticoids and glucose homeostasis

Glucocorticoids (GCs) are so named based on the results of Houssay's studies published in 1954, where it was shown that they exert an important action on glucose regulation (20) through intracellular glucocorticoid receptor protein.

Polymorphisms of glucocorticoid receptor may influence the peripheral activity of cortisol (21). In target tissues, the activation and inactivation of GCs are regulated by the enzyme 11β-hydroxysteroid dehydrogenase (22).

The GCs role on glucose homeostasis is due to the impairment of various mechanisms including β cell alterations (glucose sensitivity and insulin release) and IR, leading to a reduction in glucose disposal and to an increased production of endogenous glucose (10). Their ability to counterbalance the insulin action and to increase glucose levels are a major concern in patients with Cushing's syndrome/disease or under GCs treatment for immunomodulatory purposes (23). Physiologically, the alterations in peripheral insulin sensitivity is counterbalanced by increased β-cell function (24–27). Insulin demand is increased by GCs, and when the compensative insulin secretion is inadequate or compromised, fasting and/or postprandial hyperglycaemia may occur (10).

GCs interfere with the insulin receptor signaling cascade affecting these processes, and glucose homeostasis is usually maintained by β-cell compensations (10). Individuals with any susceptibility to glucose homeostasis alterations, such as reduced sensitivity to insulin (28), an impaired insulin response to glucose (29), first degree relatives with type 2 diabetes (30), obesity (31) and older age (32), are more prone to develop GCs induced alterations. A possible mechanism is the inadequate β-cells response. Hence, considering the negative effects of GCs on glucose homeostasis, their clinical use should be limited, especially in patients with risk factors for diabetes.

An interesting finding is that in the majority of *in vivo* studies, GCs treatment also induces an increase in glucagon and amylin plasma levels. Increased plasma amylin levels have been shown to exert a diabetogenic effect due to the higher rates of toxic amylin aggregation mediated by the islet amyloid polypeptide (IAPP) (33). Additionally, hyperglucagonaemia could oppose insulin actions and increase hepatic glucose output, a mechanism described in diabetes (34).

## GC replacement therapy in SAI

Replacement therapy in SAI does not require the mineralocorticoid administration because the renin-angiotensin-aldosterone axis is preserved (35, 36), instead of in primary adrenal insufficiency (PAI). Nevertheless, GCs replacement therapy shows wide variability in the type, dose, frequency, and timing of treatment (37).

However, there is general consent that replacement treatment should aim to obtain the physiological daily dose and to simulate the circadian cortisol rhythm.

Patients with SAI due to iatrogenic suppression of the hypothalamic–pituitary–adrenal axis requires conventional treatment and the same patient education as patients with central hypocortisolism due to disturbance involving the pituitary gland (4).

Hydrocortisone (HC), cortisone acetate (CA) and prednisone are the most frequently used drugs (37) for replacement therapy. HC and CA are usually administered with a higher dose in the morning in the fasting state and one or two fractioned doses later in the day (before 06:00 pm) (38). Compared to hydrocortisone, prednisone and dexamethasone have greater glucocorticoid activity (respectively 5 and 30- to 50-fold) and a longer half-life, exposing the patients to a greater time-exposure, and the risk of over-treatment (at the currently recommended doses), thus explaining the cardio-metabolic complications observed in these subjects (12, 39).

In patients with partial cortisol insufficiency, signs and symptoms should be evaluated to decide if the starting dose can be lower than conventional replacement doses (5–10 mg/day) or to limit exogenous glucocorticoids intake to rescue treatment when needed.

Considering a progressive worsening of the metabolic status in hypopituitary patients receiving doses greater than 20 and greater than 30 mg/day, the mean daily dose of hydrocortisone prescribed should be lower than 30 mg (40).

This is especially relevant in SAI, where many factors can influence the daily requirement of glucocorticoids, such as residual ACTH secretion, changes in binding proteins, other concomitant hormone replacements and related comorbidities.

Considering that a residual ACTH secretion and HPA-axis function may be present, lower glucocorticoid doses, especially in the afternoon, might be sufficient to replace patients with SAI when compared to PAI. Despite this, in clinical practice there is a tendency to use the same doses of GCs in both SAI and PAI, given at the same timing. It might be that in patients with SAI, even those with a non-reversible deficit, the residual ACTH secretion is sufficient to maintain the low levels that are physiologically required and produced to cover the afternoon and evening.

The modified release preparations, and the continuous infusions, has been developed to avoid the afternoon administrations, causing multiples peaks at a time when glucocorticoid sensitivity is higher, and thus replicate the shape of a gradual cortisol decline during the day of adrenal sufficient subject (15, 38). Although initially thought to treat PAI, this advantage of the modified release preparation could well apply to SAI.

## Glycometabolic effects of GC replacement therapy

### Dosing and timing

Compared to the past, the current guidelines recommend lower doses of HC to align with cortisol production rates of healthy volunteers (41). In non-AI subjects, there is a correlation between hypercortisolism and metabolic impairment, but in AI has yet to be proven (42).

An open interventional study was conducted in 17 SAI patients to evaluate the effect of increased dose of HC (or equivalent) to 30 mg/d on cardiovascular system and the role of insulin in the impairment of cardiovascular function. No significant differences in fasting and post-glucose load pulse wave velocities were observed on the higher glucocorticoid regimen. This study showed that fasting augmentation index and reactive hyperemia index were lower on the higher glucocorticoid dose and post-glucose load changes were not significantly different. The insulin sensitivity or secretion didn't show alterations on the higher glucocorticoid regimen (43).

Several studies have evaluated the effects on metabolism and glucose homeostasis of reducing the daily dose of hydrocortisone (Table 1).

**Table 1:** Studies evaluating glycometabolic effects of glucocorticoid therapeutic regimens in Secondary Adrenal Insufficiency (SAI).

**Authors**	**Study design**	**SAI/PAI/CAH**	**Patients with altered glucose homeostasis**	**Higher dose vs. lower dose of GCs**	**switch to od-MRHC**	**PAI vs. SAI**
Dunne FP (44)	Clinical trial	13/0/0	-	→ Fasting blood glucose → HbA1c → Body weight		-
McConnell EM (52)	Clinical trial	15/0/0	-	→ Insulin resistance		-
Filipsson H (40)	Observational study	1707/0/0	55/1707	↑ BMI at higher dose ↑ Waist circumference at higher dose		-
Danilowicz K (45)	Clinical trial	11/0/0	-	↓ Total body fat ↓ Abdominal fat → Fasting blood glucose → HOMA index		-
Bleicken B (47)	Observational study	140/194/0	-	→ BMI		N/A
Zueger T (48)	Retrospectivestudy	105/0/0	12/105	↑ BMI at higher dose		-
Petersons CJ (43)	Clinical trial	17/0/0	-	→ Insulin sensitivity → Insulin secretion		-
Castinetti F (46)	Observational study	122/79/0	25/201	→ Fasting blood glucose → Insulin secretion → BMI		BMI: SAI>PAI HbA1c: PAI>SAI
Quinkler M (18)	Clinical trial	18/26/6	4/50		↓ BMI ↓ HbA1c	N/A	
Mongioì LM (54)	Clinical trial	9/10/0	5/19		↓ HbA1c in PAI → HbA1c in SAI → BMI	↓ HbA1c in PAI → HbA1c in SAI
Isidori AM (19)	Randomized clinical trial	45/44/0	15/89		↓ Body weight ↓ BMI ↓ HbA1c → Fasting blood glucose → Insulin secretion → HOMA index	↓ BMI: SAI>PAI ↓ Bodyweight: SAI>PAI ↓ HbA1c: SAI = PAI
Guarnotta V (56)	Retrospective study	36/13/0	25/49		↓ BMI ↓ waist circumference ↓ HbA1c ↓ Insulin secretion ↓ Insulin AUC _2h_ ↓ VAI ↑ DIo ↑ ISI-Matsuda	↓ BMI: SAI = PAI ↓ Waist circumference:SAI = PAI ↓ HbA1c: SAI = PAI ↓ Insulin secretion: SAI = PAI ↓ Insulin AUC_2h_: SAI = PAI ↓ VAI: SAI = PAI ↑ DIo: SAI = PAI ↑ ISI-Matsuda: SAI = PAI

*SAI, secondary adrenal insufficiency; PAI, primary adrenal insufficiency; CAH, congenital adrenal hyperplasia; GCs, glucocorticoids; od-MRHC, once-daily modified-release hydrocortisone;HbA1c,glycosilated hemoglobin; BMI, body mass index; HOMAindex, homoeostatic model assessment index; AUC area under the curve; VAI, visceral adiposity index;DIo,oral disposition index; ISI-Matsuda, Matsuda index of insulin sensitivity. → no change; ↓ reduction; ↑ increase; N/A not available*.

Dunne FP et al. investigated the impact of the reduction of daily HC from 30 mg to 15 mg on cardiovascular function and body weight in 13 patients with SAI. After 3 months there was no change in body weight (44). Danilowicz et al. successively demonstrated that the reduction of HC on average by 50% in 11 patients with SAI decreased the body weight by 7 kg while insulin levels and fasting glucose unchanged (45).

In hypopituitary patients with severe untreated GHD, GC daily doses are positively correlated with BMI and serum cholesterol levels. Patients receiving HC equivalent (HCeq) doses of less than 20 mg/day did not diverge in metabolic parameters from adrenal sufficient patients. After increasing HCeq dose, higher BMI, serum lipids were observed. Baseline serum insulin growth factor I (IGF-I) varied on the basis of the type and dose of GC therapy, whereas the 1-yr response to GH treatment in metabolic risk factors was not influenced by GC regimen (40).

In a large French cohort (79 PAI, 122 SAI), Castinetti et al. found only a small difference in HC doses between patients with or with-out dyslipidemia, but not in other metabolic variables (46). In a German study (194 PAI, 140 SAI), BMI was not different in groups supplemented by high (>30 mg/day) or lower doses of HC (47). In other real-life surveys, from Poland and France, no correlation was found between the daily dose of HC and fasting glycaemia or insulinemia (46). Slightly different is the case of SAI, where an apparent relationship between weight-adjusted HC dose and high blood pressure has been described, but again not with diabetes mellitus (46, 48). Recently, a double blind, randomized, crossover trial compared the effects of 3 dosing regimens in 18 patients with SAI (49). Subjective improvement was observed with only a twice daily regimen, namely HC 10 mg at 07:00 am and 5 mg at 03:00 pm. Conversely, another Swedish study with 15 patients with PAI found that a 4-dose regimen was preferred, with higher QOL scores (50). However, this regimen resulted in a much higher cortisol exposure (24 h-cortisol area under the curve), which in the long-term might be deleterious from a metabolic point of view (50).

Recently, increased awareness about the detrimental effects of mild glucocorticoid over treatment in patients with AI has led to a lower GC daily dose. However, none of these studies found an inverse association between glucocorticoid exposure and the incidence of adrenal crises (<12.5 mg/day, 12.5–30 mg/day or >30 mg/day) (51).

McConnell et al. (52) in a randomized crossover study with 15 patients with SAI, did not find any difference in hepatic and peripheral insulin sensitivity comparing conventional oral HC replacement with HC infusions (52).

Several studies have investigated the effects of the new formulations of GCs focusing on metabolic effects of these treatments (Table 1). In particular, the od-MRHC treatment has been demonstrated to have a positive effect on body weight and glycosilated hemoglobin (HbA1_c_) in patients with PAI (17, 53). The od-MRHC regimen has been shown to slightly reduce BMI andHbA1_c_ in a previous parallel open, non-randomized, prospective trial on a mixed AI population (PAI, SAI and congenital adrenal hyperplasia) (18).

An open clinical trial evaluated the effects of od-MRHC on the glycometabolic profile and health-related quality of life of 10 PAI and 9 SAI patients. After 12 months therapy with the od-MRHC, no changes in fasting glucose, insulin resistance and BMI were observed in either population while HbA1c levels were significantly reduced in patients with PAI (54).

In a single-blind randomized active comparator, non-intervention parallel-group controlled trial, Isidori et al. (19) demonstrated that a restoration of physiological circadian glucocorticoid rhythm by replacement of conventional GC therapy with od-MRHC regimen reduced the body weight of 44 PAI and 45 SAI patients. In particular, at 24 weeks, body weight reduction was superior in patients in the od-MRHC group compared with those in the conventional treatment group (−2.1 kg [95%CI,– 4.0 to −0.3] vs.1.9 kg [– 0.1 to 3.9]; treatment difference – 4.0 kg, 95% CI −6.9 to −1.1; *p* = 0.008). Subgroup analysis for type of AI showed greater reductions in BMI (*p* = 0.081) and body weight (*p* = 0.074) in SAI than PAI patients. Conversely, any change in fasting blood glucose, insulin and the homoeostatic model assessment (HOMA) index wasn't found between the two intervention groups (conventional treatment/od-MRHC treatment) (19). This suggest that obesity in SAI can be targeted by improving GC administration.

This is consistent with the demonstration that HC administration in the evening, compared to a morning administration, produces a much more pronounced delayed hyperglycaemic effect with deleterious consequence on metabolism (55). In 1999, authors hypothesized this effect could be due to a greater sensitivity to GCs in the evening (55).

An Italian 36-months retrospective study evaluated the effects of od-MRHC in 13 PAI e 36 SAI patients with normal glucose tolerance (NGT) and pre-diabetes including impaired fasting glucose (IFG), and/or impaired glucose tolerance (IGT), on insulin secretion, and sensitivity, anthropometric parameters and cardiometabolic risk, indirectly expressed by the visceral adiposity index (VAI). In patients with AI-NGT and AI-pre-diabetes a significant decrease in BMI (*p* = 0.017 and *p* < 0.001), waist circumference (*p* = 0.008 and *p* < 0.001), HbA1c (*p* = 0.034 and *p* = 0.001) and a significant increase in high-density lipoprotein cholesterol (*p* = 0.036 and *p* = 0.043) were respectively observed. In addition, in pre-diabetic patients only a significant decrease in insulinemia (*p* = 0.014), AUC_2hinsulinemia_ (*p* = 0.038) and VAI (*p* = 0.001), in concomitance with a significant increase in the oral disposition index (DIo; *p* = 0.041) and Matsuda index of insulin sensitivity (ISI-Matsuda; *p* = 0.038) were observed. No differences in metabolic parameters, insulin secretion and sensitivity index were found comparing PAI and SAI patients (56).

## Conclusion

Secondary adrenal insufficiency has been reportedly associated with altered glycometabolic profile and increased cardiovascular risk (57), possibly due to over-replacement. However, a reduction in glucocorticoid daily dose has not been universally associated with improved glucose homeostasis, cardiovascular risk and overall morbidity. Randomized clinical trial addressing this point are very few.

In fact, specific data on glucose homeostasis alterations and improvement with different GC regimens in SAI are scarce and influenced by many confounding factors such as comorbidities, age, multiple pituitary hormone deficiencies and other replacement therapies.

In SAI patients with a residual ACTH secretion, the daily requirement of glucocorticoid might be lower than in PAI. Nevertheless, in the clinical practice, tailoring of GC replacement is often suboptimal, and there is a tendency to over-treat patients with SAI. This could be even more problematic than in PAI because of their increased metabolic risk.

Recently, the introduction of a new modified release formulation with a more physiological cortisol circadian profile has been associated with significant metabolic improvement in body weight, HbA1c and insulin sensitivity in SAI.

This prompted the hypothesis that a disruption of the physiological circadian rhythm could have a role in the observed glicometabolic alterations. Recent finding that patients with alterations of clock-related genes, such as patients with sleep disturbances or psychiatric disorders, and as recently demonstrated patients with AI (58) are exposed to increased cardiovascular risk and metabolic syndrome support this concept (59, 60).

A greater attention toward the dose and timing of glucocorticoid administration should be placed in patients with AI, tailoring the overall exposure to the residual HPA function and circadian rhythm of affected patients in order to limit their adverse metabolic profile.

## Author contributions

CG, VH, MV, DG, AI, and ES have equally contributed to the paper, the literature review, analysis, interpretation, drafting, and editing for the final submission.

### Conflict of interest statement

CG and AI have received honoraria from Shire and Novartis for speaking or serving on advisory boards and grants. ES has received honoraria from Shire for speaking or serving on advisory boards and grants. AI and DG have received honoraria for speaking and serving on advisory boards from Novartis, IPSEN, Otsuka, and menarini. The remaining authors declare that the research was conducted in the absence of any commercial or financial relationships that could be construed as a potential conflict of interest.
